# Vienna Letter

**Published:** 1890-04

**Authors:** Hugh Hagan

**Affiliations:** Vienna, Austria


### VIENNA LETTER.

VIENNA, AUSTRIA, February 23rd, 1890.

*To the Southern Medical Record.*

Since I wrote last, "La Grippe" has made its third appearance in Vienna. Although it was less general, yet many victims were found, and the symptoms were even severer than earlier in the history of its appearance.

The after-effects of a second or third attack are frightful Among the severest and yet most frequent, are such conditions as severe anæmia, multiple peripheral neuritis, loss of appetite, and a general debility, that takes good food, much alcohol, and rest with gentle exercise, quite two or three weeks to restore the patient to his former condition.

In looking over the death rate of the general hospital, some days since, I was surprised to find that of the post-mortems made in the Pathological Institute, about 4,000 yearly, in 70 per cent. of the case, some forms of tuberculosis was found, and on further questioning Professor Kundrath, he stated that over 33 per cent. of the cases were brought to an end by tuberculosis. This is a frightful condition of affairs, and the sanitary authorities are now trying to combat this terrible scourge. On asking Professor Kundrath what he thought the possible cause of this large percentage of tuberculosis, he said, "it was most possibly made worse than it would otherwise be, by the poor diet the working classes were obliged to live upon. The average daily wages of laborers is about 30 to 40 cents a day. This hardly gives them house rent and black bread, for meat is almost unknown to them."

I was told by a very worthy old gentleman, an Austrian by birth, that the health of Vienna was, or had been for the past year, giving the Board of Health great concern, and that, as a result of their investigations, a system of public kitchens had been established. The object of these kitchens is to furnish, at very moderate prices, food that meets the requirements of a healthy physiology. This organization has met with great success, and the pathologists all seem to think, that the frightful percentage of tuberculosis will be lessened, by giving to the poor, bodies that have sufficient vitality to resist the invasion of this, mankind's most subtle and dangerous enemy.

There has been organized in Vienna a Society called "The Anglo-American Vienna Medical Association," whose objects and aims are to render to any Englishman or American the necessary information in regard to the requirements of the medical school. The society will forward to any applicant, its catalogue, in which are contained every advice needed. The want of such an institution has long been felt by the medical students. They were formerly obliged to spend a good deal of time and money before they were able to get completely in the swing, but under the guidance of this catalogue, the stranger can, in two or three days, get to work, thus saving much valuable time and worry. The society's address is, 12 Landesgerichts Strasse, Vienna.

Charcot's method of suspension in Tabesdorsalis is practiced only to a very limited extent in Vienna, and so far as I have seen, only by Dr. Benedict, of the Polyclinic. In the course of the last five months attendance on this clinic, I have seen the Professor suspend only three cases. The patients, one a young woman of 22, who had been suffering for 6 years, and was entirely unable to walk, even with crutches; the other two were men about 40 to 55 years respectively. They were both unable to walk without aid, and could with difficulty go, with the aid of their two sticks. Without exception, the ataxic symptoms were greatly relieved, and indeed to such an extent, that the patients were able to walk and stand alone. The oldest of the patients, who suffered frightfully from lancinating pains, was generally relieved, and his symptoms were much better for three or four days.

The manner of suspension is as follows: Dr. Sayre's apparatus for suspension is the one used, and only made very strong. The chin rest, and axillary pads are adjusted so that the weight shall be equally distributed, and the patient is raised about a foot in the air, and allowed to remain suspended about three to five minutes. If the patient complains of pressure or faint, he must be immediately lowered, and the suspension deferred until another day.

These three patients experience such benefit that regularly once or twice a week, they come to be suspended. On asking the Professor as to the permanent effects over the advancement of the sclerotic process, he was doubtful as to any.

But while he finds no rational ground for the suspension, yet he states that if the empricism be found harmless, and comfort can be elicited, why practice suspension.

I do not know whether Charcot's suspension in Tabes suggested suspension in paralysis agitans to Dr. Benedict or not; but I have seen very marked effects on two cases of paralysis agitans suspended by Dr. Benedict. The patients, both men, one 70 odd, and the other 80, were sufferers from the above mentioned disease; and to marked degrees. The manner of suspension was the same as that practiced in tabes, only the old men could not stand over three minutes. In both cases, were the tremors much improved, and the patients stated, that for some days were they much more at rest.

We had a very interesting case of "Acromagalia," at the Benedict clinic. The case was an interesting one, for more reasons than one, first on account of its rarity, and secondly, if I be not mistaken, first mentioned by Dr. Hammond, of Washington, D. C. The patient, a woman 34 years, unmarried, and who had for the past three years suffered from amenorrhœa. Her genitalia were prematurely atrophied. She had never born children. The patient stated that up to the time of her irregularity in her menses she had enjoyed good health, and was not of the same type woman, formerly. She had been a large woman, but well made and features well cut. The picture the patient presented was as follows:

A woman of about 5 feet, 8 inches tall, forehead a normal form and shape, face somewhat enlarged, but not in proportion to the nose and lower jaw. The development of the nose and chin gave the face a most masculine, gigantic and dull expression. The hands were fully four times the size of an ordinary hand, and much puffed. The bony frame looked as though an Hercules would have been proud to have claimed them as "his own." The whole picture looked supramundane. The woman was of fair intelligence and complaimed only of the amenorrhœa, and this gigantic growth. She looked somewhat sluggish, but talked very well, and gave quite a complete history of the case. The muscular strength of this woman was strikingly in contrast with the picture she presented. Her grip was not equal to that of an ordinary working woman.

Our small Anglo-American colony of doctors are as busy as a hive of bees. Now is just the season that the old ones are leaving for home, or other parts, such as Paris or London, while the new ones, fresh from college or hospital, are pouring in, eager for the fray, and almost envious of those who are ready to start on the road to fame, charged with English and Austrian medical lore. HUGH HAGAN, M. D.

